# Associations Between XPD Lys751Gln Polymorphism and Leukemia: A Meta-Analysis

**DOI:** 10.3389/fgene.2018.00218

**Published:** 2018-06-14

**Authors:** Min Wen, Bo Zhou, Xin Lin, Yunhua Chen, Jialei Song, Yanmei Li, Eldad Zacksenhaus, Yaacov Ben-David, Xiaojiang Hao

**Affiliations:** ^1^State Key Laboratory of Functions and Applications of Medicinal Plants, Guizhou Medical University, Guiyang, China; ^2^College of Basic Medicine, Guizhou Medical University, Guiyang, China; ^3^The Key Laboratory of Chemistry for Natural Products of Guizhou Province, Chinese Academy of Sciences, Guiyang, China; ^4^Department of Medicine, Toronto General Research Institute, University Health Network, Toronto, ON, Canada

**Keywords:** leukemia, XPD, ERCC2, meta-analysis, polymorphism

## Abstract

**Objectives:** The aim of the present study was to define the potential relationship between xeroderma pigmentosum group D (XPD) Lys751Gln polymorphisms and the risk of leukemia.

**Methods:** A comprehensive search of Pubmed, Web of Science, EBSCO, the Cochrane Library and China National Knowledge Infrastructure was conducted to identify original articles published before March 2017 concerning the association between XPD Lys751Gln polymorphisms and leukemia risk. A literature quality assessment was performed using the Newcastle-Ottawa Scale. Heterogeneity across studies was assessed using *I*^2^ statistics. Random- or fixed-effects models were used to calculate pooled odds ratios (ORs) in the presence or absence of heterogeneity, respectively. Sensitivity analysis was used to assess the influence of individual studies on the pooled estimate. Publication bias was investigated using funnel plots and Egger’s regression test. All data analyses were performed using Stata 14.0 and Revman 5.3.

**Results:** Fourteen studies with a total of 7525 participants (2,757 patients; 4,768 controls) were included in this meta-analysis. We found that XPD Lys751Gln polymorphisms significantly increased the risk of developing leukemia in both dominant OR = 1.21, 95%CI [1.10–1.35], *P* ≤ 0.001) and heterozygote (OR = 1.22, 95%CI [1.09–1.36], *P* ≤ 0.001) model. An allele model showed a borderline significant increase in leukemia risk (OR = 1.13, 95%CI [1.00–1.27], *P* = 0.05). A subgroup analysis revealed a consistent association between XPD Lys751Gln polymorphisms and leukemia risk for some genetic models in Caucasian populations, adult or chronic groups, and in almost all models of childhood or acute groups.

**Conclusion:** Our results indicate that XPD Lys751Gln polymorphism increases the risk of leukemia, especially in childhood and acute cases.

## Introduction

Leukemia, a common malignant disease of the hematopoietic system (Jiang et al., 2014), can be classified on the basis of speed of disease progression and cell cytogenetics into four common subtypes: acute myeloid leukemia (AML), acute lymphoblastic leukemia (ALL), chronic myeloid leukemia (CML) and chronic lymphocytic leukemia (CLL) (Arber et al., 2016). The etiology and mechanisms underlying of leukemogenesis are still unclear, although radiation, smoking, obesity and exposure to chemical carcinogens are considered high risk factors (Larsson and Wolk, 2008; Fircanis et al., 2014; Malagoli et al., 2016; Nikkila et al., 2016). Nevertheless, only a small proportion of people exposed to these risks develops leukemia, suggesting that hereditary factors may play a critical role in leukemia carcinogenesis (Li et al., 2016; Huang and Ovcharenko, 2017).

Decreased efficiency of DNA repair is considered a crucial event in carcinogenesis (Hoeijmakers, 2001). Recently, a series of studies revealed that reduced DNA repair, leading to chromosomal aberrations and genomic instability, is a major contributor to the pathogenesis of leukemia (Das-Gupta et al., 2000; Esposito and So, 2014). The xeroderma pigmentosum group D (XPD) gene, also known as ERCC2, encodes a 5′–3′ superfamily 2 helicase that plays a key role in unwinding the DNA double helix around damaged DNA during nucleotide excision repair (NER) (Kuper et al., 2012; Constantinescu-Aruxandei et al., 2016). Because of the biological significance of XPD, XPD polymorphisms have been extensively studied in different malignant diseases, such as pancreatic (Wu et al., 2017), colorectal (Ni et al., 2014) and gallbladder (Srivastava et al., 2010) cancers.

Several studies have reported inconsistent results on the relationship between the XPD Lys751Gln polymorphism (SNP IDs: rs13181) and leukemia susceptibility. Some studies showed a clear relationship between XPD Lys751Gln polymorphism and an increased risk of leukemia (Juan et al., 2005; Ganster et al., 2009; Shi et al., 2011; Banescu et al., 2014, 2016), while others showed a decreased risk of leukemia (Ozcan et al., 2011; Douzi et al., 2015), and others suggested no association between this polymorphism and leukemia (Seedhouse et al., 2002; Allan et al., 2004; Matullo et al., 2006; Mehta et al., 2006; Pakakasama et al., 2007; Batar et al., 2009; Canalle et al., 2011; Ozdemir et al., 2012; Sorour et al., 2013; Dincer et al., 2015). To evaluate more precisely the potential relationship between XPD Lys751Gln polymorphism and leukemia, we hereby report a meta-analysis using all the available published data.

## Materials and Methods

### Search Strategies

A computerized search of Pubmed, Web of Science, EBSCO, the Cochrane Library and China National Knowledge Infrastructure (CNKI) up to March 2017 was conducted using the following search strategy: (“XPD” or “ERCC2” or “xeroderma pigmentosum group D”), and (“polymorphism” or “variant” or “mutation”), and “Leukemia.” The search was restricted to English and Chinese language publications. A manual search of the references in the retrieved articles and relevant reviews was also conducted. A flowchart of information pertaining to identification, screening, eligibility, and the final selected datasets was constructed in accordance with the Preferred Reporting Items for Systematic Reviews and Meta-analyses (PRISMA) guidelines (Moher et al., 2009).

### Inclusion and Exclusion Criteria

Studies that investigated the association between XPD Lys751Gln polymorphism and leukemia risk were included. The inclusion criteria were (1) case-control study design; (2) available genotype information of the XPD Lys751Gln polymorphism; (3) evaluation of the XPD gene polymorphism and the risk of leukemia; and (4) the distribution of genotypes among the controls was in agreement with the Hardy–Weinberg equilibrium (HWE). The major criteria for exclusion were: (1) duplication of earlier publications (for studies using the same sample in different publications, only the most complete information was included following careful examination), (2) unpublished papers, dissertations, conference articles and reviews, (3) family-based studies of pedigrees.

### Data Extraction

Data from each eligible study were extracted into Excel, and included the country of origin and ethnicity of each study population, age group (adult or childhood), subtypes of leukemia, genotyping method, numbers of cases and controls, numbers of cases and controls in the XPD Lys751Gln genotypes and results of the HWE test.

### Study Quality Assessment

The quality of the included studies was assessed by two reviewers in accordance with the Newcastle-Ottawa Scale (NOS) (Stang, 2010), which is used to assess the quality of observational studies. Discrepancies were reported and settled by a third party. Three major aspects of study quality were scored: (1) selection of the study groups (0 ± 4 points); (2) determination of the exposure of interest in the studies (0 ± 3 points); and (3) the quality of the adjustment for confounding variables (0 ± 2 points). A study could be scored with a maximum of one star for each item numbered within the categories of Selection and Exposure, while at most two stars could be allocated to Comparability. A higher score represents a greater quality of the study methodology. A score equal to or higher than 6 was considered to indicate high study quality.

### Data Analysis

The combined odds ratios (ORs) and 95% confidence interval (CI) were used to evaluate the strength of the association with the risk of leukemia. Pooled ORs were performed for allelic comparison (a vs. A), dominant (aa + Aa vs. AA), recessive (aa vs. Aa + AA) and codominant (aa vs. AA and Aa vs. AA) models (“a” and “A” represents the mutant allele and the wild-type allele, respectively). Heterogeneity among the included studies was assessed using the *I*^2^ statistic. A random-effects model or fixed-effects model was used to calculate the pooled OR in the presence or absence of heterogeneity, respectively. To detect possible sources of heterogeneity and potential differences among subgroups, meta-regression and subgroup analyses were carried out with the stratification of different ethnicities, age groups and subtypes of leukemia. The significance of the pooled OR was determined through a *Z*-test, and *p* < 0.05 was considered to be statistically significant. Publication bias was investigated using funnel plots and Egger’s regression test. We also conducted sensitivity analysis to test the robustness of associations by sequentially omitting each of the included studies one at a time. All the data analysis was performed using STATA software 14.0 (StataCorp, College Station, TX, United States) and Review Manager 5.3 (Cochrane Collaboration, Oxford, United Kingdom).

## Results

### Literature Search and Study Characteristics

**FIGURE 1:**
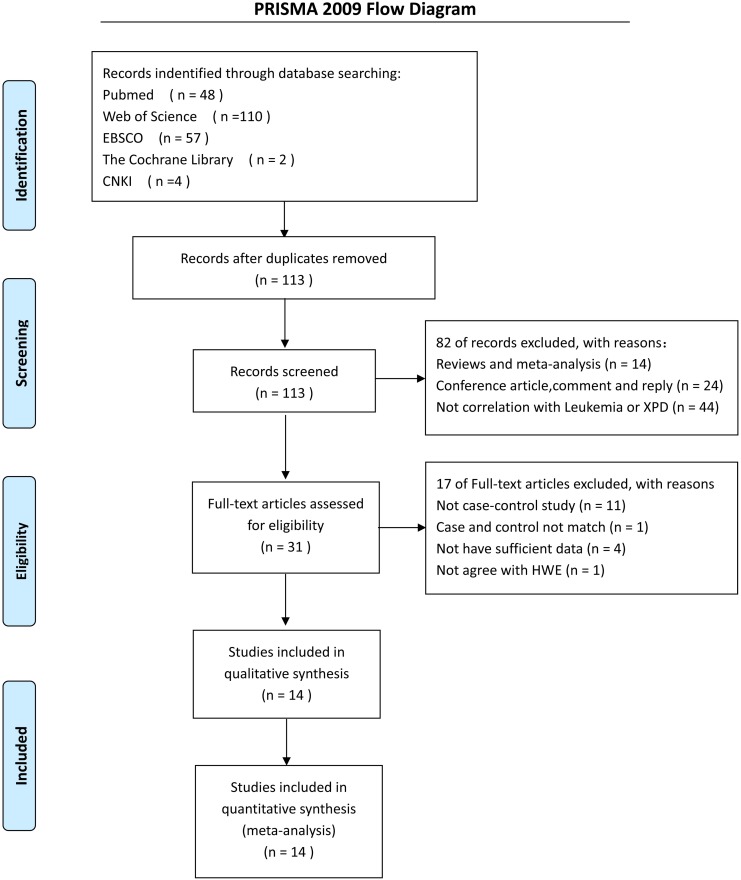
A flow diagram showing the study selection process for the meta-analysis.

We used several search criteria to include or exclude reported studies on the relationship between XPD polymorphism and leukemia (**Figure 1**). A total of 14 studies (2,757 cases and 4,768 controls) concerning XPD Lys751Gln polymorphism were included in the final evaluation (**Table 1**). A quality assessment of the individual studies showed that the Newcastle-Ottawa scale score ranged from 6 to 8, indicating that the quality of the methodology was generally good (**Table 2**).

**Table 1:** Main characteristics of datasets included for the XPD Lys751Gln polymorphism and leukemia risk.

Author	Year	Country	Ethnicity	Subtypes	Age group	Genotyping method	Sample size		Genotype (n/frequency)						HWE
									Case			Control			
									
							Case	Control	Lys/Lys	Lys/Gln	Gln/Gln	Lys/Lys	Lys/Gln	Gln/Gln	
Seedhouse	2002	United Kingdom	Caucasians	AML	Adult	PCR-RFLP	122	73	44 36.07%	59 48.36%	19 15.57%	30 41.10%	32 43.83%	11 15.07%	0.6195
Allan	2004	United Kingdom	Caucasians	AML	Adult	PCR-RFLP	474	696	180 37.97%	216 45.57%	78 16.46%	293 42.10%	299 42.96%	104 14.94%	0.0597
Matullo	2006	European	Caucasians	Unclassified	Adult	TaqMan	169	1094	70 41.42%	79 46.75%	20 11.83%	397 36.29%	504 46.07%	193 17.64%	0.1330
Mehta	2006	United States	Caucasians	AML	Child	TaqMan	313	432	116 37.06%	142 45.37%	55 17.57%	183 42.36%	194 44.91%	55 12.73%	0.7521
			African	AML	Child	TaqMan	38	146	20 52.63%	18 47.37%	0 0%	87 59.59%	52 35.62%	7 4.79%	1.0000
Pakakasama	2007	Thailand	Asia	ALL	Child	PCR-RFLP	108	317	87 80.56%	19 17.59%	2 1.85%	260 82.02%	56 17.67%	1 0.31%	0.4932
Batar	2009	Turkish	Caucasians	ALL	Child	PCR-RFLP	70	75	26 37.14%	33 47.14%	11 15.72%	27 36.00%	35 46.67%	13 17.33%	0.8119
Ganster	2009	Austria	Caucasians	CLL	Adult	PCR-RFLP	444	444	157 35.36%	222 50.00%	65 14.64%	186 41.89%	194 43.69%	64 14.42%	0.2589
Canalle	2011	Brazil	Caucasians	ALL	Child	PCR-RFLP	162	223	72 44.44%	78 48.15%	12 7.41%	105 47.09%	101 45.29%	17 7.62%	0.3415
			African	ALL	Child	PCR-RFLP	27	138	16 59.26%	9 33.33%	2 7.41%	91 65.94%	40 28.99%	7 5.07%	0.4134
Ozcan	2011	Turkish	Caucasians	AML	Adult	PCR-RFLP	35	100	18 51.43%	16 45.71%	1 2.86%	41 41.00%	41 41.00%	18 18.00%	0.2049
				ALL	Adult	PCR-RFLP	10	100	4 40.00%	4 40.00%	2 20.00%	41 41.00%	41 41.00%	18 18.00%	
Shi JY	2011	China	Asia	AML	Adult	MALDI-TOF MS	303	554	243 80.20%	58 19.14%	2 0.66%	480 86.64%	70 12.64%	4 0.72%	0.3373
Sorour A	2013	Egypt	Caucasians	AML	Adult	PCR-RFLP	90	60	33 36.67%	45 50.00%	12 13.33%	27 45.00%	30 50.00%	3 5.00%	0.2197
Banescu C	2014	Romania	Caucasians	CML	Adult	PCR-RFLP	156	180	51 32.69%	77 49.36%	28 17.95%	82 45.56%	79 43.89%	19 10.55%	1.0000
Douzi K	2015	Tunisia	Caucasians	ALL	No age limit	PCR-RFLP	85	206	33 38.82%	43 50.59%	9 10.59%	92 44.66%	93 45.15%	21 10.19%	0.8742
				AML		PCR-RFLP	34	206	21 61.77%	11 32.35%	2 5.88%	92 44.66%	93 45.15%	21 10.19%	
				CML		PCR-RFLP	87	206	40 45.98%	46 52.87%	1 1.15%	92 44.66%	93 45.15%	21 10.19%	
Dincer Y	2015	Turkish	Caucasians	ALL	Child	PCR-RFLP	30	30	11 36.67%	12 40.00%	7 23.33%	9 30.00%	17 56.67%	4 13.33%	0.4721

*AML, Acute myeloid leukemia; ALL, acute lymphoid leukemia; CLL, chronic lymphocytic leukemia; CML, chronic myeloid leukemia. PCR-RFLP, polymerase chain reaction-restriction fragment length polymorphism; MALDI-TOF MS, matrix assisted laser desorption/ionization time-of-flight mass spectrometry. HWE P-value in the control group, P > 0.05 indicates that the participants in the control group met the HWE.*

**Table 2:** Quality assessment analysis.

Number	Author	Selection	Exposure	Comparability	Total score
1	Seedhouse C	4	2	2	8
2	Allan JM	3	2	1	6
3	Matullo G	4	2	1	7
4	Mehta PA	3	2	1	6
5	Pakakasama S	3	2	1	6
6	Batar B	4	2	2	8
7	Ganster C	4	2	2	8
8	Canalle R	4	2	1	7
9	Ozcan Ali	4	2	1	7
10	Shi JY	3	2	1	6
11	Sorour A	4	2	1	7
12	Banescu C	4	2	2	8
13	Douzi K	3	2	2	7
14	Dincer Y	4	2	2	8

### Association Between the XPD Lys751Gln Polymorphism and Risk of Leukemia

**FIGURE 2:**
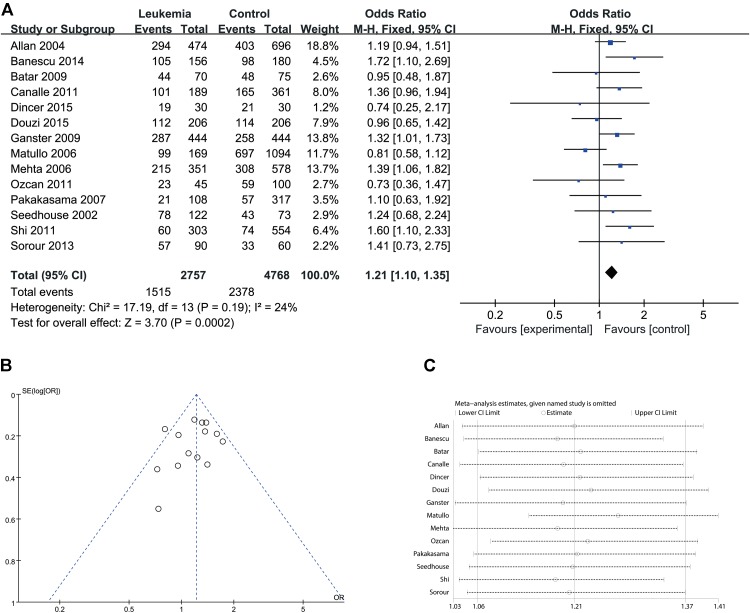
Comparison of XPD Lys751Gln for overall data in dominant model (Gln/Gln + Lys/Gln vs. Lys/Lys). **(A)** Forest plot, **(B)** funnel plot, **(C)** sensitivity analysis.

**FIGURE 3:**
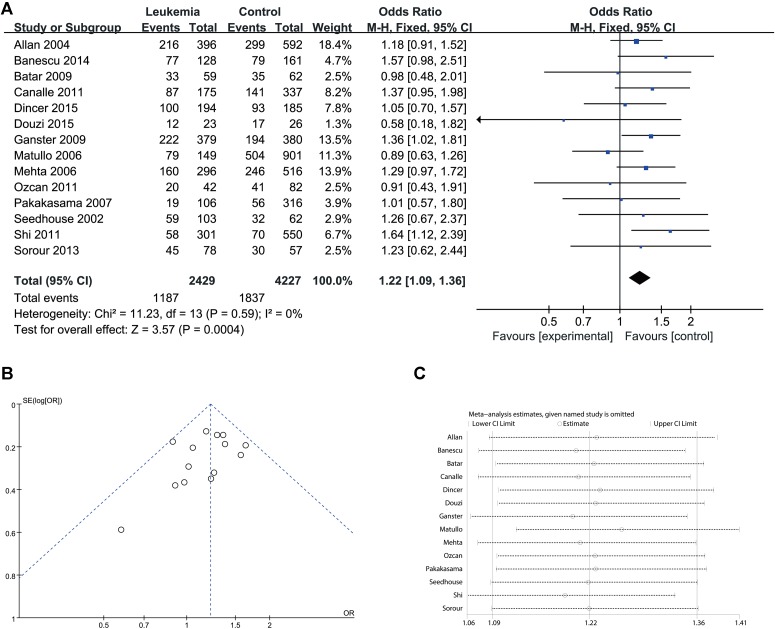
Comparison of XPD Lys751Gln for overall data in heterozygote model (Lys/Gln vs. Lys/Lys). **(A)** Forest plot, **(B)** funnel plot, **(C)** sensitivity analysis.

**FIGURE 4:**
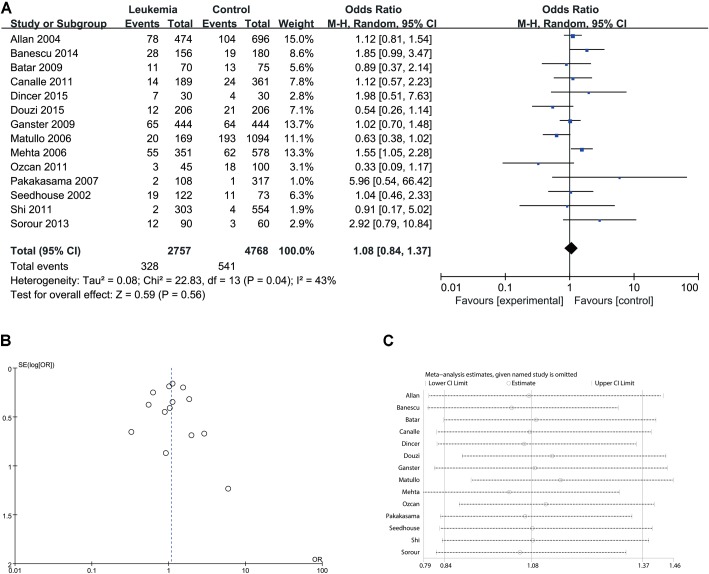
Comparison of XPD Lys751Gln for overall data in recessive model (Gln/Gln vs. Lys/Gln + Lys/Lys). **(A)** Forest plot, **(B)** funnel plot, **(C)** sensitivity analysis.

**FIGURE 5:**
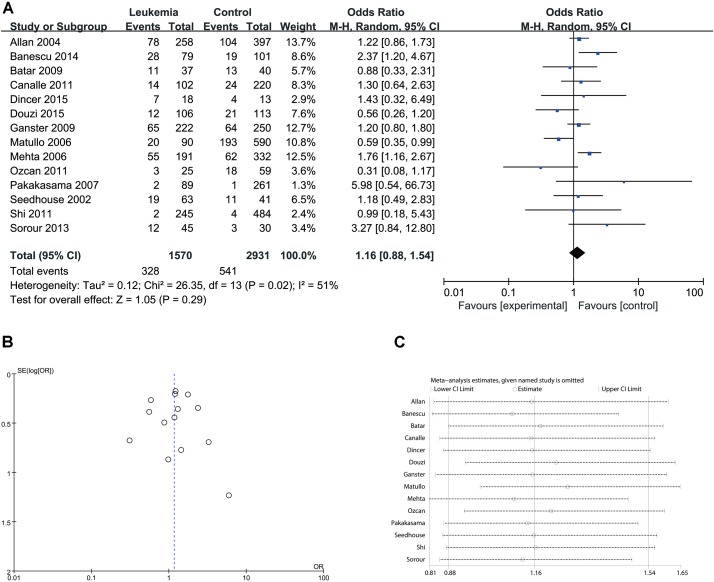
Comparison of XPD Lys751Gln for overall data in homozygote model (Gln/Gln vs. Lys/Lys). **(A)** Forest plot, **(B)** funnel plot, **(C)** sensitivity analysis.

**FIGURE 6:**
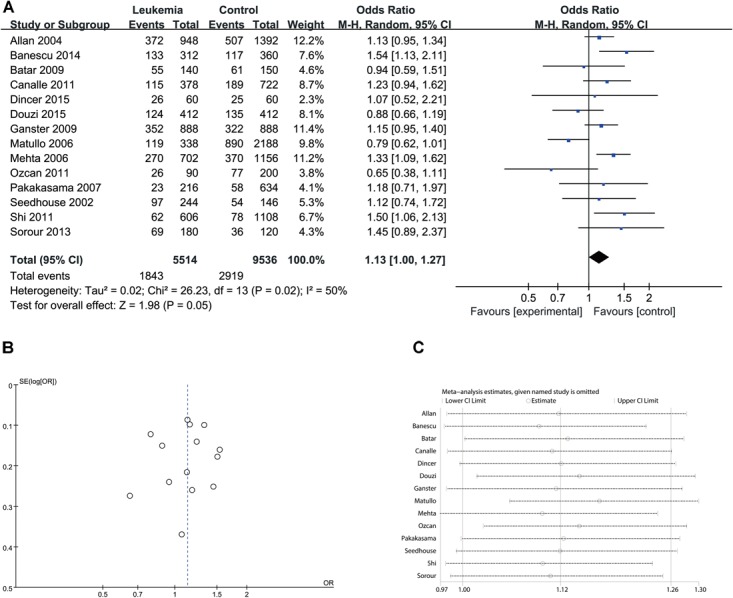
Comparison of XPD Lys751Gln for overall data in allele model (Gln vs. Lys). **(A)** Forest plot, **(B)** funnel plot, **(C)** sensitivity analysis.

Since significant heterogeneity was identified in recessive, homozygote and alleles models, a random-effects model was used. The other genetic models were analyzed using a fixed-effects model. Overall, a significant increase in leukemia risk was identified in dominant (Gln/Gln + Lys/Gln vs. Lys/Lys: *I*^2^ = 24%, *P* < 0.001, **Figure 2**) and heterozygote models (Lys/Gln vs. Lys/Lys: *I*^2^ = 0%, *P* < 0.001, **Figure 3**). No significant association was found in recessive (Gln/Gln vs. Lys/Gln + Lys/Lys: *I*^2^ = 43%, *P* = 0.560, **Figure 4**) and homozygote (Gln/Gln vs. Lys/Lys: *I*^2^ = 51%, *P* = 0.29, **Figure 5**) models. In addition, the allele model showed a borderline significant increase in leukemia risk (Gln vs. Lys: *I*^2^ = 50%, *P* = 0.05, **Figure 6**). Moderate heterogeneity (*I*^2^: 43%–51%) was found in the no-association model group. To explore the source of this heterogeneity, a meta-regression analysis was conducted. The results revealed that the heterogeneity was not associated with ethnicity, age, clinical subtype or detection method (*p* ≥ 0.05 in all genetic models). We further explored the source of heterogeneity by removing one study each time. The results showed that Matullo et al. (2006) was one of the central sources of the heterogeneity (its inclusion increased heterogeneity by 12%–24%). No publication bias was found in any of the models. Sensitivity analysis suggested that with exception of the allele model, the results were stable and reliable.

### Association Between the XPD Lys751Gln Polymorphism and Risk of Leukemia by Ethnicity

In Caucasian populations, a significant increase in leukemia risk was found in the heterozygote model (Lys/Gln vs. Lys/Lys: *I*^2^ = 0%, *P* = 0.02) and dominant models (Gln/Gln + Lys/Gln vs. Lys/Lys: *I*^2^ = 14%, *P* = 0.02). No significant association was found in recessive (Gln/Gln vs. Lys/Gln + Lys/Lys: *I*^2^ = 44%, *P* = 0.75), homozygote (Gln/Gln vs. Lys/Lys: *I*^2^ = 55%, *P* = 0.32) and allele models (Gln vs. Lys: *I*^2^ = 47%, *P* = 0.24) (**Table 3**). In African and Asian populations, the subgroup analysis was unreliable as only two studies were available.

**Table 3:** Summary of pooled OR in different ethnicities.

Genetic model	Age group	Pooled OR (95% CI)	Heterogeneity		Test for overall effect		Statistical model
					
			*P*	*I*^2^	*Z*	*P*	
Gln vs. Lys	Caucasians	1.08 [0.95–1.21]	0.04	47%	1.18	0.24	Random-effects model
	African	1.16 [0.74–1.82]	0.66	0%	0.63	0.53	Fixed-effects model
	Asian	1.39 [1.05–1.86]	0.45	0%	2.26	0.02	Fixed-effects model
GlnGln vs. LysLys	Caucasians	1.16 [0.87–1.56]	0.01	55%	1.00	0.32	Random-effects model
	African	0.85 [0.21–3.38]	0.29	12%	0.23	0.82	Fixed-effects model
	Asian	1.77 [0.48–6.52]	0.23	30%	0.86	0.39	Fixed-effects model
LysGln vs. LysLys	Caucasians	1.15 [1.02–1.30]	0.77	0%	2.34	0.02	Fixed-effects model
	African	1.41 [0.81–2.48]	0.78	0%	1.20	0.23	Fixed-effects model
	Asian	1.35 [0.86–2.14]	0.17	46%	1.30	0.19	Random-effects model
GlnGln + LysGln vs. LysLys	Caucasians	1.15 [1.02–1.28]	0.31	14%	2.39	0.02	Fixed-effects model
	African	1.33 [0.77–2.30]	1.00	0%	1.02	0.31	Fixed-effects model
	Asian	1.42 [1.04–1.94]	0.27	17%	2.24	0.03	Fixed-effects model
GlnGln vs. LysGln + LysLys	Caucasians	1.04 [0.81–1.33]	0.05	44%	0.32	0.75	Random-effects model
	African	0.75 [0.19–2.93]	0.26	22%	0.41	0.68	Fixed-effects model
	Asian	1.67 [0.46–6.14]	0.21	36%	0.78	0.44	Fixed-effects model

### Association Between the XPD Lys751Gln Polymorphism and Risk of Leukemia by Age

In a subgroup analysis by age group, one study was excluded as it lacked data on patients’ age (Douzi et al., 2015). Significant associations were consistently found in some genetic models of the adult group (GlnGln + LysGln vs. LysLys: *I*^2^ = 46.0%, *P* = 0.04; LysGln vs. LysLys: *I*^2^ = 9%, *P* = 0.002), and in almost all models of the childhmodels of the childhood group (Gln vs. Lys: *I*^2^ = 0%, *P* = 0.003; GlnGln + LysGln vs. LysLys: *I*^2^ = 0%, *P* = 0.01; GlnGln vs. LysGln + LysLys: *I*^2^ = 0%, *P* = 0.03; GlnGln vs. LysLys: *I*^2^ = 0%, *P* = 0.009; LysGln vs. LysLys: *I*^2^ = 0%, *P* = 0.04). Moderate to higher heterogeneity (*I*^2^: 46%–65%) was found in the no-association models of the adult group (**Table 4**).

**Table 4:** Summary of pooled OR in different age groups.

Genetic model	Age group	Pooled OR (95% CI)	Heterogeneity		Test for overall effect		Statistical model
					
			*P*	*I*^2^	*Z*	*P*	
Gln vs. Lys	Adult	1.13 [0.95–1.35]	0.006	65%	1.37	0.17	Random-effects model
	Childhood	1.24 [1.08–1.43]	0.74	0%	2.97	0.003	Fixed-effects model
GlnGln vs. LysLys	Adult	1.13 [0.77–1.66]	0.02	58%	0.64	0.52	Random-effects model
	Childhood	1.54 [1.11–2.13]	0.54	0%	1.08	0.28	Fixed-effects model
LysGln vs. LysLys	Adult	1.24 [1.08–1.42]	0.36	9%	3.06	0.002	Fixed-effects model
	Childhood	1.21 [1.01–1.45]	0.77	0%	2.05	0.04	Fixed-effects model
GlnGln+LysGln vs. LysLys	Adult	1.22 [1.01–1.48]	0.07	46%	2.04	0.04	Random-effects model
	Childhood	1.28 [1.06–1.54]	0.65	0%	2.57	0.01	Fixed-effects model
GlnGln vs. LysGln + LysLys	Adult	1.03 [0.76–1.41]	0.07	47%	0.19	0.85	Random-effects model
	Childhood	1.40 [1.04–1.90]	0.51	0%	2.19	0.03	Fixed-effects model

### Association Between the XPD Lys751Gln Polymorphism and Risk of Leukemia by Subtype

In a subgroup analysis by leukemia subtypes, one study was excluded as it lacked such data (Matullo et al., 2006). Significant associations were found in almost all genetic models of acute leukemia (Gln vs. Lys: *I*^2^ = 12%, *P* ≤ 0.001; GlnGln + LysGln vs. LysLys: *I*^2^ = 0%, *P* ≤ 0.001; GlnGln vs. LysLys: *I*^2^ = 14%, *P* = 0.02; LysGln vs. LysLys: *I*^2^ = 0%, *P* = 0.003), and in some models of chronic disease (GlnGln + LysGln vs. LysLys: *I*^2^ = 34%, *P* = 0.009; LysGln vs. LysLys: *I*^2^ = 0%, *P* ≤ 0.001) (**Table 5**).

**Table 5:** Summary of pooled OR in different leukemia subtype.

Genetic model	Subtype	Pooled OR (95% CI)	Heterogeneity		Test for overall effect		Statistical model
					
			*P*	*I*^2^	*Z*	*P*	
Gln vs. Lys	Acute	1.17 [1.07–1.29]	0.33	12%	3.37	0.0007	Fixed-effects model
	Chronic	1.14 [0.83–1.57]	0.03	72%	0.80	0.42	Random-effects model
GlnGln vs. LysLys	Acute	1.29 [1.04–1.59]	0.32	14%	2.35	0.02	Fixed-effects model
	Chronic	1.08 [0.40–2.88]	0.01	78%	0.15	0.88	Random-effects model
LysGln vs. LysLys	Acute	1.22 [1.07–1.39]	0.76	0%	2.99	0.003	Fixed-effects model
	Chronic	1.36 [1.09–1.69]	0.67	0%	2.70	0.007	Fixed-effects model
GlnGln + LysGln vs. LysLys	Acute	1.24 [1.09–1.40]	0.63	0%	3.38	0.0007	Fixed-effects model
	Chronic	1.32 [1.07–1.63]	0.22	34%	2.60	0.009	Fixed-effects model
GlnGln vs. LysGln + LysLys	Acute	1.19 [0.98–1.45]	0.35	10%	1.72	0.09	Fixed-effects model
	Chronic	0.94 [0.39–2.28]	0.02	76%	0.13	0.90	Random-effects model

## Discussion

The NER pathway is a highly conserved DNA repair mechanism that removes bulky intra-strand adducts created by agents such as UV radiation and certain chemicals, including several commonly used chemotherapy agents (Scharer, 2013). Genetic polymorphism in DNA repair genes may cause variation in DNA repair capacity, which in turn can lead to cumulative genotoxic damage and increased susceptibility to cancer (Douzi et al., 2015). As an important component of NER, XPD is an evolutionarily conserved ATP-dependent DNA helicase that plays an essential role in DNA repair (Kim et al., 2016). Several studies have suggested that individuals with polymorphisms in XPD or other NER pathway genes may have an increased risk of cancer (Paszkowska-Szczur et al., 2013; He et al., 2016). Moreover, several molecular epidemiological studies have found an association between XPD polymorphism and leukemia risk in diverse populations. However, the results are inconsistent and even contradictory. We therefore conducted a meta-analysis to globally evaluate the potential relationship between XPD polymorphism and leukemia.

Our results indicate that XPD Lys751Gln polymorphism significantly increases the overall leukemia risk in dominant and heterozygote models, but not in an allele model or homozygote model. The results suggest that heterozygous mutations but not homozygote mutations of XPD (Lys/Gln) may increase genetic susceptibility to leukemia. This may be the result of a higher rate of heterozygous versus homozygous mutations (the ratio between heterozygous and homozygous mutations is 2.27 ∼ 56 in the control and 1.71 ∼ 29 in leukemia). A subgroup analysis by ethnicity showed the same result in a Caucasian population. The exact mechanism underlying the association between the susceptibility to different tumors and XPD Lys751Gln polymorphism is currently unknown. XPD is a 5′–3′ superfamily 2 DNA helicase that opens damaged DNA for bulky lesion repair in NER. The interaction of the C-terminal domain of XPD with the p44 helicase activator protein is critical for both helicase activity and the stability of the Transcription Factor II H (TFIIH) complex, which is essential for RNA polymerase II-mediated transcription initiation and the NER (Liu et al., 2008). The XPD C-terminal Lys751Gln polymorphism may alter the structure of the C-terminal domain, there by blocking critical interaction with p44 and creating a destructive TFIIH conformation, which subsequently reduces DNA repair activity (Lunn et al., 2000; Fan et al., 2008; Monaco et al., 2009). Furthermore, by stratifying the data by age and disease subtype, we found that XPD Lys751Gln polymorphism significantly increased leukemia risk in almost all models of childhood and acute disease. The occurrence and development of leukemia appeared to be regulated by genetic and environmental factors. In children with acute leukemia, malignancy manifests with a short latency period, and therefore there is not enough exposure time to allow the initiation of a long carcinogenic process. Unlike children, adults usually develop cancer because of the cumulative effect of environmental exposure during their life (Brisson et al., 2015). Therefore, we speculate that genetic polymorphism is more important for childhood and acute leukemia.

Compared with previous reports (Liu et al., 2014; Wu et al., 2014), the present study has the following advantages: (1) it analyzed the association between XPD Lys751Gln polymorphism and both acute and chronic leukemia; (2) a total of 14 studies (2,757 cases and 4,768 controls) were included in this analysis, there by increasing the statistical power of the analysis. (3) Strict literature inclusion and exclusion criteria were enforced, the Ozdemir’s study was excluded from our analysis because of a lack of discerning information between ALL and Burkitt lymphoma patients. However, our research also has some limitations: (1) the number of original studies included in the meta-analysis is relatively small, especially in the subgroup analysis. Future studies on ethnicity, age and subtype of leukemia are needed to further corroborate our findings. (2) Heterogeneity, which can greatly affect the conclusions of meta-analyses, was high in some models. Our results showed that moderate or higher heterogeneity was found in some models, which showed no association with leukemia risk. Matullo et al. (2006) was a major source of this heterogeneity, probably to because of the significant differences in the number of cases and controls.

## Conclusion

Our meta-analysis demonstrates that XPD Lys751Gln polymorphism significantly increases overall leukemia risk in dominant and heterozygote models, and that this polymorphism is significantly associated with almost all genetic models of childhood and acute leukemia.

## Author Contributions

XH and Y-BD designed this study. XL, YC, and JS searched databases and collected the full-text papers. MW and BZ extracted and analyzed the data and wrote the manuscript. EZ, YL, XH, and Y-BD reviewed the manuscript.

## Conflict of Interest Statement

The authors declare that the research was conducted in the absence of any commercial or financial relationships that could be construed as a potential conflict of interest.
